# Analysis and Accuracy Improvement of UWB-TDoA-Based Indoor Positioning System

**DOI:** 10.3390/s22239136

**Published:** 2022-11-24

**Authors:** Paolo Grasso, Mauro S. Innocente, Jun Jet Tai, Olivier Haas, Arash M. Dizqah

**Affiliations:** 1 Autonomous Vehicles & Artificial Intelligence Laboratory (AVAILAB), Centre for Future Transport and Cities, 7th Floor Friars House, Manor House Drive, Coventry CV1 2TE, UK; 2 Centre for Future Transport and Cities, 7th Floor Friars House, Manor House Drive, Coventry CV1 2TE, UK; 3 Smart Vehicles Control Laboratory (SVeCLab), University of Sussex, Brighton BN1 9RH, UK

**Keywords:** IPS, ultra-wideband, time difference of arrival, Cramér–Rao lower bound, CRLB, bifurcation curve, debiasing, filtering

## Abstract

Positioning systems are used in a wide range of applications which require determining the position of an object in space, such as locating and tracking assets, people and goods; assisting navigation systems; and mapping. Indoor Positioning Systems (IPSs) are used where satellite and other outdoor positioning technologies lack precision or fail. Ultra-WideBand (UWB) technology is especially suitable for an IPS, as it operates under high data transfer rates over short distances and at low power densities, although signals tend to be disrupted by various objects. This paper presents a comprehensive study of the *precision*, *failure*, and *accuracy* of 2D IPSs based on UWB technology and a pseudo-range multilateration algorithm using Time Difference of Arrival (TDoA) signals. As a case study, the positioning of a $4\times 4\;m^{2}$ area, four anchors (transceivers), and one tag (receiver) are considered using bitcraze’s Loco Positioning System. A Cramér–Rao Lower Bound analysis identifies the convex hull of the anchors as the region with highest *precision*, taking into account the anisotropic radiation pattern of the anchors’ antennas as opposed to ideal signal distributions, while bifurcation envelopes containing the anchors are defined to bound the regions in which the IPS is predicted to *fail*. This allows the formulation of a so-called *flyable area*, defined as the intersection between the convex hull and the region outside the bifurcation envelopes. Finally, the static bias is measured after applying a built-in Extended Kalman Filter (EKF) and mapped using a Radial Basis Function Network (RBFN). A debiasing filter is then developed to improve the *accuracy*. Findings and developments are experimentally validated, with the IPS observed to *fail* near the anchors, *precision* around ±3cm, and *accuracy* improved by about 15cm for static and 5cm for dynamic measurements, on average.

## 1. Introduction

Positioning that is accurate and precise as well as robust and reliable has become an essential part of many applications which require determining the position of an object in space, such as monitoring the location of assets, people, and goods and assisting navigation systems with varying degrees of autonomy while operating within potentially complex and dynamic environments [1,2]

A variety of positioning systems exist which make use of different (i) *technologies*, (ii) *signal properties*, and (iii) *positioning algorithms*. *Technologies* include inertial navigation systems (INS) [3,4], sound waves [5,6], infrared [7], visible light [8], and radio frequency, including Ultra-Wide Band (UWB) [9], Bluetooth [10], Bluetooth Low Energy (BLE) [11], ZigBee, Wireless Local Area Network (WLAN) [12,13], and Wireless Underground Sensor Network [14]. *Signal properties* used for positioning include Angle of Arrival (AoA) [7], Time of Arrival (ToA) [14], Time Difference of Arrival (TDoA), Received Signal Strength Indication [15], Time of Flight (ToF), Return Time of Flight (RToF), and Phase of Arrival (PoA) [2]. *Positioning algorithms*, which include triangulation, trilateration [16,17], proximity, and Two-Way Ranging algorithms [18], are then used in conjunction with the aforementioned *technologies* and *signal properties* to estimate and/or track the position of an object. Positioning algorithms tend to be overly sensitive to external disturbances, and therefore often rely on sensor fusion. While this is most often a form of Kalman filter, machine learning models such as neural networks [19,20], clustering algorithms [21], or Bayesian models [22,23] can be used.

Global Navigation Satellite Systems (GNSS) are suitable for efficient outdoor long-range positioning. While the most common technology is the Global Positioning System (GPS), the European Galileo started providing services in 2016 with a constellation of 26 satellites [24]. A GNSS allows an electronic receiver to determine its position by trilateration using radio signal travel times (ToA) from at least four satellites [25]. However, because these signals cannot penetrate walls or objects, use of this technology for Indoor Positioning Systems (IPSs) is infeasible. Conversely, UWB technology is well-suited for IPSs, as they are characterised by large bandwith, high data transfer rates over short distances, short message length, low transmission power, and high obstacle penetration capability [1,9]. In addition, UWB-based IPSs constitute one of the most accurate and precise positioning technologies at present, and are arguably the best choice among current technology [9,26] despite their susceptibility to interference caused by metallic materials or systems working on similar frequencies. Considering the recent drive towards autonomy and self-organisation in robotics (e.g., [27,28]), the precision and accuracy of the IPS is crucial for performing indoor experiments efficiently and safely.

Traditionally, the precision of positioning systems has been studied by performing a Cramér–Rao Lower Bound (CRLB) analysis [29] from the signals perspective, then applying coefficients such as the Geometric Dilution of Precision (GDoP) [30] to capture the geometrical features, e.g., to identify where there is a sudden drop in IPS performance. Examples include the calculation of bounds for ToA [31,32], anchor time synchronisation [33,34,35], and AoA [36]. Such work is of critical importance, as the performance of UWB-based IPSs tends not to be homogenous across the measurement domain [37]; thus, characterising its performance is crucial to understanding the limits of the system and potentially optimising its design and/or layout [38].

CRLB analysis is widely accepted for positioning systems in which the tagged object to be localised/tracked is not in close proximity to the anchors; in such scenarios, localisation algorithms tend to fail due to *flipping uncertainty*, a well-known problem of geometrical origin [39,40]. Regions in the measurement domain where failure occurs due to geometrical constraints are not considered in the CRLB analysis [38,41]. Furthermore, the possibility of signal degradation due to the anisotropic signal transmission properties of the anchors is almost never considered, even though it is an important property that many antenna optimisation studies have accounted for [42,43,44,45].

This paper is concerned with UWB-based IPSs aimed at localising a tagged moving object (receiver) based on the spatial distribution of the anchors (transceivers) using pseudorange multilateration algorithm and signal TDoA measurements. More specifically, it is concerned with the planar localisation performance within a homogeneous medium with negligible interference and reverberation, while taking into account the anisotropic radiation pattern of an actual DWM1000 antennae module rather than simply assumming ideal signal distributions. A rigorous analysis of system performance is carried out in terms of *precision*, *failure*, and *accuracy*, informed by previous work reported in [38,39,40,46,47,48,49,50]. To summarise, the contributions of this paper are as follows:

(i) A theoretical study of the *precision* of the position estimates is performed based on a CRLB analysis for round-robin scheduling and an anisotropic representation of the signal-to-noise ratio function of the 3D radiation pattern of the anchor antennas.
(ii) A geometrical study of the 2D IPS domain is carried out, defining bifurcation envelopes that bound the areas where the IPS is predicted to fail. This complements the CRLB analysis, which does not predict regions of failure. Together, they define the so-called *flyable area* in which positioning is reliable.
(iii) Experiments using an existing IPS with four anchors and a static tagged object are used to validate the *precision* and *failure* predictions and to estimate the bias (*inaccuracy*).
(iv) A debiasing filter is developed to increase the *accuracy* of the static position estimates, which is then tested for both static and moving tagged objects.

The remainder of this paper is organised as follows: Section 2 presents a comprehensive study of the *precision* and *failure* of IPSs based on UWB technology and a pseudorange multilateration algorithm using signal TDoA, including a description of the IPS under study in Section 2.1, a theoretical study of precision using CRLB analysis in Section 2.2, and a geometrical study of failure using a bifurcation envolope analysis in Section 2.3. Section 3 proposes a process to measure the accuracy of the IPS and a debiasing filter to improve it. Section 4 presents the design of the validation and testing experiments, with the results discussed in Section 5. Finally, our conclusions are drawn in Section 6.

## 2. Theoretical Study of Precision and Failure

The purpose of an IPS is to localise indoor tagged objects (receivers) using the spatial distribution of anchors at known locations (transceivers). The aim here is to study the *precision* and *failure* of the system. The former is studied using a CRLB analysis that is specific for TDoA algorithms with round-robin scheduling, while a study of the bifurcation envelope is carried out to identify areas where the system is expected to fail.

### 2.1. IPS under Study

**Figure 1:**
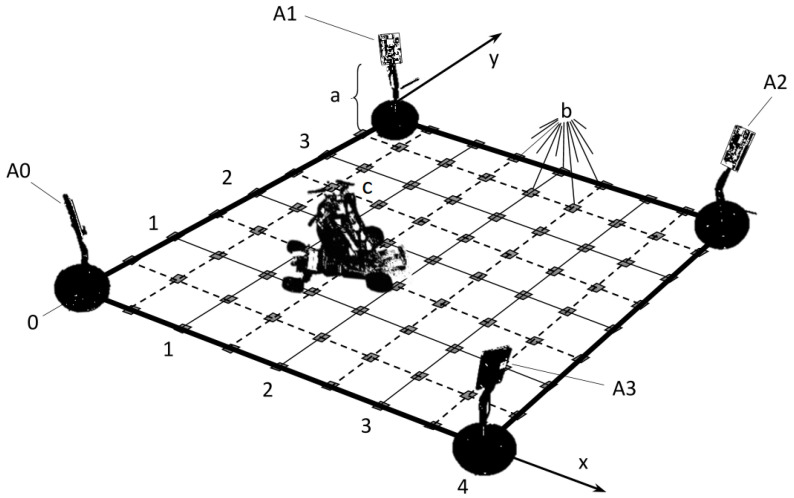
Not-to-scale diagram of the 4×4 m^2^ 2D IPS being studied: (a) adjustable stands for the transmitting anchor antennas (A0–A3); (b) measurement points distributed every 50 cm in each direction; and (c) mobile stand for the object to be localised.

**Figure 2:**
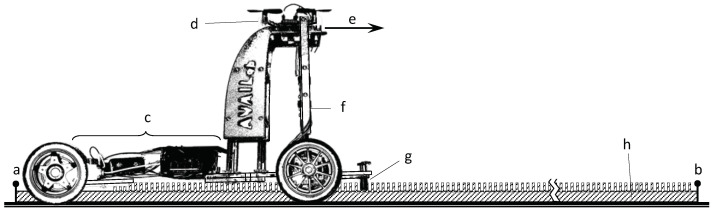
Dynamic experiment setup: mobile stand on rail; (a,b) beginning and end of rail; (h) optical obstacles (pins); (g) optical infrared sensor; (c) pcu, batteries, and motors; (d) Crazyflie 2.0; (e) direction of movement; (f) laser pointer. Optical sensor aligned with drone’s antenna.

The system to be studied uses bitcraze’s Loco Positioning System [51] and consists of a drone to be localised and four transceiver anchors positioned at the vertices and facing the centre of a $4\times 4\;m^{2}$ domain, as shown in Figure 1. All antennas under study are at a height of 20 cm above the floor; the drone is mounted on a sliding ground-referenced measurement system parallel to the floor equipped with a laser pointer aligned with the onboard UWB antenna to achieve reference positioning with high precision (±1 mm) and accuracy (see Figure 2). This experimental setup is used in Section 4 and Section 5, where the regularly spaced markers on the floor are the sampling positions to be used.

It is important to note that this work assumes that the tagged object to be localised acts strictly as a passive receiver, whereas in the literature it is often a transceiver. Nonetheless, the theoretical results are applicable to both cases as long as the receivers are sensitive and approximately omnidirectional.

### 2.2. CRLB Analysis for Pseudo-Range Multilateration with Round-Robin Scheduling

The Cramér–Rao Lower Bound (CRLB) analysis is generally deemed suitable for evaluating the precision of an unbiased IPS. It is based on the concept of the Fisher Information Matrix (FIM) for the likelihood of obtaining a correct measurement. For more details on the theory and terminology, refer to Appendix B. The elements of the total FIM for the general positioning problem [48,52] are as shown in Equation (1).

(1) $${\mathrm{FIM}}_{ij}={\left(\frac{\partial h(x)}{\partial x_{i}}\right)}^{T}F_{\tau }^{-1}\left(x\right)\left(\frac{\partial h(x)}{\partial x_{j}}\right)+\frac{1}{2}tr\left(F_{\tau }^{-1}\left(x\right)\frac{\partial F_{\tau }\left(x\right)}{\partial x_{i}}F_{\tau }^{-1}\left(x\right)\frac{\partial F_{\tau }\left(x\right)}{\partial x_{j}}\right)$$

where h(x) is the range vector (e.g., distance between receiver and anchors), $F_{\tau }$ is the covariance matrix of the $\hat{\tau }$ measurements, and tr(·) is the trace function. Equation (1) considers that the standard deviations ($\sigma_{i}$) of the likelihood function (and hence $F_{\tau }$) vary in space. One column of the Jacobian matrix of h is defined as in Equation (2).

(2) $$\frac{\partial h(x)}{\partial x_{i}}=\left[\begin{array}{c} \frac{\partial h_{12}\left(x\right)}{\partial x_{i}}\\ \frac{\partial h_{23}\left(x\right)}{\partial x_{i}}\\ \frac{\partial h_{34}\left(x\right)}{\partial x_{i}}\\ \frac{\partial h_{41}\left(x\right)}{\partial x_{i}} \end{array}\right]$$

TDoA measurements for *N* anchors and round-robin scheduling are referred to as $\tau_{rr}=\left\{\tau_{12},\tau_{23},\ldots ,\tau_{N1}\right\}$. Thus, the divergence matrix of h for $\tau_{rr}=\left\{\tau_{12},\tau_{23},\tau_{34},\tau_{41}\right\}$ for the TDoA2 protocol used by the Loco Positioning System [51,53] is as in Equation (3).

(3) $$\frac{\partial h(x)}{\partial x}={\left[\begin{array}{cc} \frac{x-x_{1}}{∥x-x_{1}∥}-\frac{x-x_{2}}{∥x-x_{2}∥} & \frac{y-y_{1}}{∥x-x_{1}∥}-\frac{y-y_{2}}{∥x-x_{2}∥}\\ \frac{x-x_{2}}{∥x-x_{2}∥}-\frac{x-x_{3}}{∥x-x_{3}∥} & \frac{y-y_{1}}{∥x-x_{1}∥}-\frac{y-y_{2}}{∥x-x_{2}∥}\\ \frac{x-x_{3}}{∥x-x_{3}∥}-\frac{x-x_{4}}{∥x-x_{4}∥} & \frac{y-y_{3}}{∥x-x_{3}∥}-\frac{y-y_{4}}{∥x-x_{4}∥}\\ \frac{x-x_{4}}{∥x-x_{4}∥}-\frac{x-x_{1}}{∥x-x_{1}∥} & \frac{y-y_{4}}{∥x-x_{4}∥}-\frac{y-y_{1}}{∥x-x_{1}∥} \end{array}\right]}_{4\times 2}$$

Making use of the linear properties of the expected value, the diagonal elements of $F_{\tau }$ can be calculated as in Equation (4), and its connected elements for consecutive estimators as in Equation (5), where only the $j^{\mathrm{th}}$ anchor is in common; the hat identifies measurements, the bar stands for the mean, and E[·] stands for expectation.

(4) Fij,ij=E(τ^ij−τ¯ij)2=E((τ^i−τ¯i)−(τ^j−τ¯j))2=E(τ^i−τ¯i)2+E(τ^j−τ¯j)2−2E(τ^i−τ¯i)(τ^j−τ¯j)=σi2+σj2

(5) $$\begin{array}{c} F_{ij,jk}=E\left[\left({\hat{\tau }}_{ij}-{\bar{\tau }}_{ij}\right)\left({\hat{\tau }}_{jk}-{\bar{\tau }}_{jk}\right)\right]=\\ E\left[\left(\left({\hat{\tau }}_{i}-{\bar{\tau }}_{i}\right)-\left({\hat{\tau }}_{j}-{\bar{\tau }}_{j}\right)\right)\left(\left({\hat{\tau }}_{j}-{\bar{\tau }}_{j}\right)-\left({\hat{\tau }}_{k}-{\bar{\tau }}_{k}\right)\right)\right]=\\ E\left[-{\left({\hat{\tau }}_{j}-{\bar{\tau }}_{j}\right)}^{2}\right]=-E\left[{\left({\hat{\tau }}_{j}-{\bar{\tau }}_{j}\right)}^{2}\right]=-\sigma_{j}^{2} \end{array}$$

Estimating the covariance between seemingly uncorrelated TDoA measurements (${\hat{\tau }}_{ij}$, ${\hat{\tau }}_{kp}$) is not trivial. From the Cauchy–Bunyakovsky–Schwarz inequality, we can derive Equation (6) where $\Delta {\hat{\tau }}_{ij}=\left({\hat{\tau }}_{ij}-{\bar{\tau }}_{ij}\right)$.

(6) $$\begin{array}{c} {\left(E[\Delta {\hat{\tau }}_{ij}\cdot \Delta {\hat{\tau }}_{kp}]\right)}^{2}⩽E\left[\Delta {\hat{\tau }}_{ij}^{2}\right]\cdot E\left[\Delta {\hat{\tau }}_{kp}^{2}\right]\Rightarrow \\ -\sqrt{E\left[\Delta {\hat{\tau }}_{ij}^{2}\right]\cdot E\left[\Delta {\hat{\tau }}_{kp}^{2}\right]}⩽E\left[\Delta {\hat{\tau }}_{ij}\cdot \Delta {\hat{\tau }}_{kp}\right]⩽\sqrt{E\left[\Delta {\hat{\tau }}_{ij}^{2}\right]\cdot E\left[\Delta {\hat{\tau }}_{kp}^{2}\right]} \end{array}$$

From Equations (5) and (6), the covariance between ${\hat{\tau }}_{ij}$ and ${\hat{\tau }}_{kp}$ can be bounded as shown in Equation (7).

(7) $$\begin{array}{c} 0>F_{ij,kp}=E\left[\left({\hat{\tau }}_{ij}-{\bar{\tau }}_{ij}\right)\left({\hat{\tau }}_{kp}-{\bar{\tau }}_{kp}\right)\right]\geq -\sqrt{E\left[{\left({\hat{\tau }}_{ij}-{\bar{\tau }}_{ij}\right)}^{2}\right]\cdot E\left[{\left({\hat{\tau }}_{kp}-{\bar{\tau }}_{kp}\right)}^{2}\right]}=\\ -\sqrt{\left(\sigma_{i}^{2}+\sigma_{j}^{2}\right)\left(\sigma_{k}^{2}+\sigma_{p}^{2}\right)}. \end{array}$$

Thus, the information matrix of the TDoA measurements set $\tau_{rr}=\left\{\tau_{12},\tau_{23},\tau_{34},\tau_{41}\right\}$ for four coplanar anchors using an efficient unbiased estimator is provided by the measurement covariance matrix in Equation (8), where $s_{i}$ stands for $\sigma_{i}^{2}$.

(8) $$\begin{array}{c} F_{\tau }={\left[\begin{array}{cccc} s_{1}+s_{2} & -s_{2} & F_{12,34} & -s_{1}\\ -s_{2} & s_{2}+s_{3} & -s_{2} & F_{23,41}\\ F_{12,34} & -s_{3} & s_{3}+s_{4} & -s_{4}\\ -s_{1} & F_{23,41} & -s_{2} & s_{4}+s_{1} \end{array}\right]}_{4\times 4}\\ F_{12,34}=-\sqrt{\left(s_{1}+s_{2}\right)\left(s_{3}+s_{4}\right)}\\ F_{23,41}=-\sqrt{\left(s_{2}+s_{3}\right)\left(s_{4}+s_{1}\right)} \end{array}$$

Kaune et al. [48] suggest that the variance for a specific source is as in Equation (9):

(9) $$\begin{array}{c} \sigma_{i}^{2}\left(r\right)=\left\{\begin{array}{cc} \frac{a}{{\mathrm{SNR}}_{0}}\cdot \frac{r_{i}^{2}}{r_{0}^{2}} & \mathrm{if}\;r_{i}\geq r_{0}\\ \frac{a}{{\mathrm{SNR}}_{0}} & \mathrm{if}\;r_{i}<r_{0} \end{array}\right.\\ \mathrm{with}\;a=\frac{c^{2}}{B^{2}} \end{array}$$

where SNR_0_ is the *signal-to-noise* power ratio at a threshold distance $r_{0}$ from the $i^{\mathrm{th}}$ anchor under consideration, *c* is the signal propagation speed, and *B* is the bandwidth of the received signal. The SNR_0_ varies with the view angle θ if the antenna has some directionality. In order to evaluate the SNR(x), we use the *Friis formula* for noise, which provides the relation between the signal gain (over noise) and distance between the transmitter and receiver for different channel frequencies.

#### 2.2.1. Signal-to-Noise Ratio Formulation

The SNR in Equation (10) is the ratio between the power of the signal reaching the receiver ($P_{r}$) and the power of the noise ($P_{N}$):

(10) $$\mathrm{SNR}\left(d,\theta_{t},\phi_{t},f_{\mathrm{ref}},T,P_{t}\right)=\frac{P_{r}}{P_{N}}$$

It can be expressed as a function of the distance between transmitter and receiver (*d*), the representative transmission frequency ($f_{\mathrm{ref}}$) and bandwidth of the selected channel, the temperature of the environment (*T*), the transmitting power ($P_{t}$), and the gains of both the transmitting antenna ($G_{t}$) and the receiving antenna ($G_{r}$). Because the receiving antenna is usually very sensitive, $G_{r}$ may be neglected in this analysis. The gain $G_{t}$ can be a function of the azimuth ($\theta_{t}$) and elevation ($\phi_{t}$) angles with respect to the frame of reference centered on the antenna. The power at the end of the transmission line can be expressed using the contemporary Friis law, as shown in Equation (11):

(11) $$P_{r}=\frac{P_{t}\cdot G_{t}\cdot G_{r}}{L_{t}\cdot L_{r}}\cdot {\left(\frac{c}{4\pi \cdot f_{\mathrm{ref}}\cdot d}\right)}^{2}$$

where $L_{t}$ and $L_{r}$ are the electric losses in the electronics of the transmitter and receiver modules, respectively, which have been embedded in the gains $G_{t}$ and $G_{r}$. Here, it is convenient to express everything in logarithmic form.

Combining Equations (9) and (11), the upper bound of the standard deviation is obtained as in Equation (12), where dBm stands for dB milli-watts; $P_{t\mathrm{dBm}}\left(T,V_{i}\right)$ is an experimental curve approximating the relationship between the transmission power, the ambient temperature, and the input voltage ($V_{i}$), as in Equation (13) [54]; and $G_{t\mathrm{dBi}}\left(\theta_{t},\phi_{t}\right)$ is the measured transmitting antenna gain with respect to an isotropic antenna, which is a 3D radiation pattern function of the azimuth and elevation angles [55]. Note that the noise power is expanded into a thermal noise power term, $k_{B}TB_{w}$, where $k_{B}$ is the Boltzmann constant for radiation of a black body (≈$1.38\times 10^{-23}J/K$).

(12) $$\left\{\begin{array}{c} \begin{array}{c} {\mathrm{SNR}}_{\mathrm{dB}}=P_{t\mathrm{dBm}}\left(T,V_{i}\right)+G_{t\mathrm{dBi}}\left(\theta_{t},\phi_{t}\right)-10\log_{10}\left(k_{B}TB_{w}10^{3}\right)-20\log_{10}\left(4\pi f_{\mathrm{ref}}d/c\right) \end{array}\\ \sigma^{2}=\frac{c^{2}}{B_{w}^{2}}\cdot 10^{-{\mathrm{SNR}}_{\mathrm{dB}}/10} \end{array}\right.$$

(13) $$P_{t\mathrm{dBm}}\left(T,V_{i}\right)=P_{t0\mathrm{dBm}}+{\left.\frac{\partial P_{t}}{\partial T}\right|}_{T_{\mathrm{ref}}}\left(T-T_{\mathrm{ref}}\right)+{\left.\frac{\partial P_{t}}{\partial V}\right|}_{V_{\mathrm{ref}}}\left(V_{i}-V_{\mathrm{ref}}\right)$$

#### 2.2.2. Radiation Pattern of the DW1000 Anchor Antenna

Most theoretical studies tend to use ideal distributions of the signal around the antennae (isotropic, bi-conical, cardioid, unidirectional, etc.); however, we hypothesise that the actual signal distribution is important, as it may have a non-negligible impact on the precision of the IPS.

**Figure 3:**
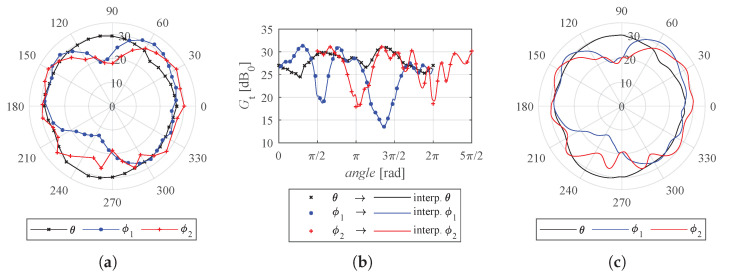
(**a**) Original experimental radiation pattern sections on the θ, $\phi_{1}$, and $\phi_{2}$ planes; (**b**) approximation procedure forcing identical values on intersections; and (**c**) radial projection of the approximated radiation pattern sections. These are used to reconstruct the 3D radiation pattern.

**Figure 4:**
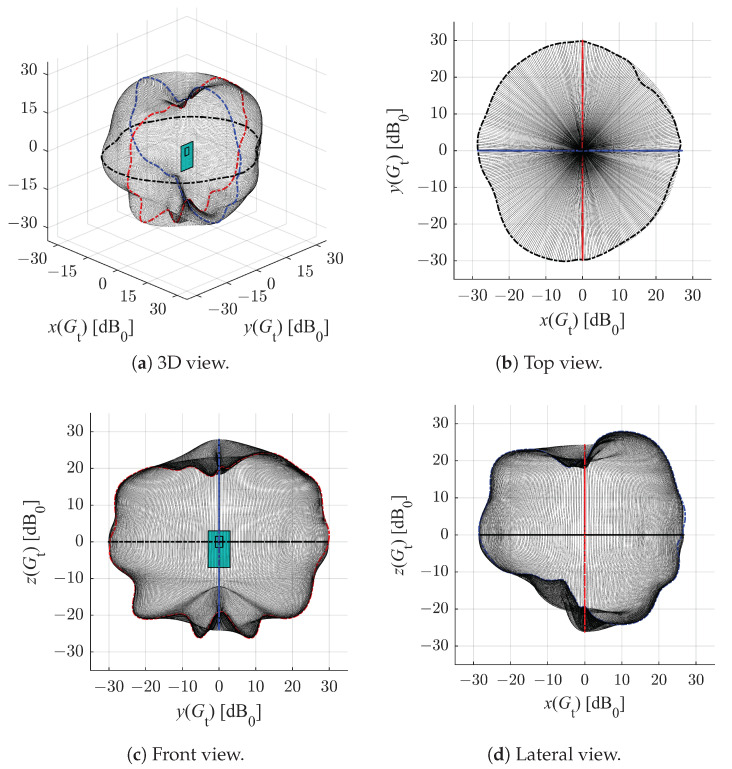
Views of the reconstructed 3D radiation pattern of an anchor antenna.

In order to reconstruct the 3D radiation pattern from the three measured sections in the azimuth (θ), elevation-1 ($\phi_{1}$) and elevation-2 ($\phi_{2}$) planes (see Figure 3), we formulate a linear combination of the boundary values of the considered quadrant. Using the system of Equations (14), the 3D radiation pattern depicted in Figure 4 can be obtained.

(14) $$\left\{\begin{array}{c} a_{1}=cos^{2}\left(\theta \right)\cdot \left(1-cos^{40}\left(\phi \right)\right)\\ a_{2}=\left(1-cos^{2}\left(\theta \right)\right)\cdot \left(1-cos^{40}\left(\phi \right)\right)\\ a_{3}=cos^{40}\left(\phi \right)\\ G\left(\theta ,\phi \right)=a_{1}\cdot G_{\phi_{1}}+a_{2}\cdot G_{\phi_{2}}+a_{3}\cdot G_{\theta } \end{array}\right.$$

The obtained radiation pattern of the antennas supports our choice to place the anchors in our experiments facing the centre of the domain (see Section 2.1), despite BitCraze seemingly recommending that they be placed in pairs facing each other and forming a 90-degree angle with the floor (see figure with eight anchors placed in a room in [56]). Furthermore, this study provides additional variables for the optimal design problem formulation of IPSs, namely, the antenna orientations.

#### 2.2.3. Analytical Results of CRLB Analysis

**Figure 5:**
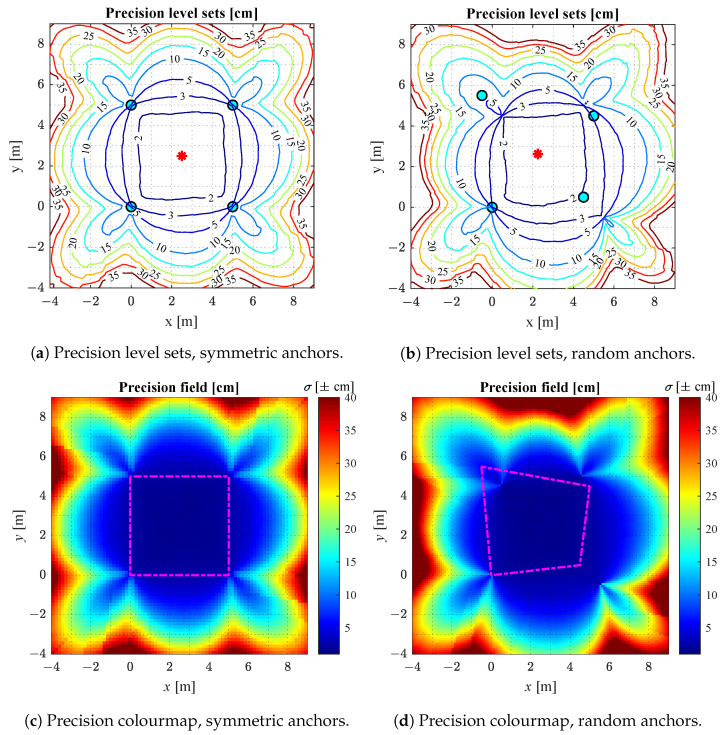
Precision level sets and colourmaps for symmetric and random anchors. The magenta trapezoid is the convex hull of four anchors (modified from [38], with permission).

The CRLB analysis carried out here considers two different representative distributions of four anchors, namely, a symmetrical layout and a random one, as shown in Figure 5. The ripples of the contour lines in Figure 5 are to be expected due to the anisotropy of the radiation pattern in Figure 4.

The best precision is obtained within the convex hull of the anchors. In this case, it is about ±5 cm with 99% confidence level (i.e., k=2.58). A realistic non-isotropic transmitting antenna gain (DWM1000 module [55]) is applied for the estimation of the SNR, hence the slight fluctuations in the represented values.

### 2.3. Bifurcation Envelope Analysis

The CRLB analysis is crucial for estimating the *precision* of the positioning system across the domain of measurements, although the analysis does not consider regions in the measurement domain where *failure* occurs due to geometrical constraints [38,41]. For instance, IPSs suffer from a well-known problem of geometrical origin called *flipping uncertainty* [39,40]. A TDoA map, which is a geometrical representation of the TDoA measurements, can be used to address this issue. Thus, we define a so-called *flyable area* using a combination of the CRLB analysis carried out in Section 2.2 and the bifuraction envelope derived from a geometrical study carried out in this section. Specifically, this *flyable area* defines a region of space within which system precision is guaranteed to be inside the bounds calculated using the CRLB analysis. It follows that any object’s location measured should only be trusted when within this domain.

#### 2.3.1. Bifurcation Curve

**Figure 6:**
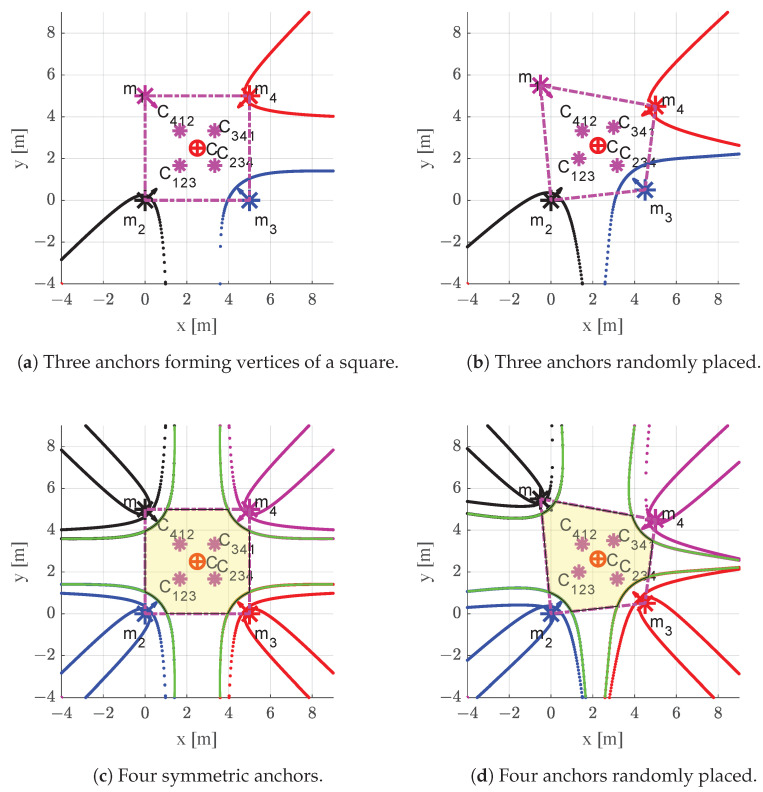
Bifurcation curves, bifurcation envelopes (green line), convex hull of anchors (magenta trapezoid, acceptable precision), and flyable area (yellow shade) for three and four anchors.

The bifurcation curve is the projection of the TDoA map boundaries from the τ-plane (pseudo-range space) to the space of source localisation (2D in this case). The bifurcation curve, as defined in [39], is the quintic curve $\tilde{E}\left(x\right)$ depicted by the roots of a polynomial P(x) which represents the TDoA map constraints. The definition of P(x) and examples of algebraic equations of $\tilde{E}\left(x\right)$ can be found in [47], while its rigorous derivation is presented in [39] using tools such as exterior algebra formalism and Minkowski space. This formulation is invariant under permutation of the TDoA measurements, which means that scheduling does not affect this analysis. Any TDoA-based system has a unique solution of the positioning problem if P(x) is negative, which defines the region outside the bifurcation curves surrounding the anchors. The multilateration algorithm within the bifurcation curves (convex regions) returns either two mirrored solutions or complex solutions with no physical meaning. An example of a bifurcation curve is shown in Figure 6a,b for the case of three anchors {$m_{2},m_{3},m_{4}$}.

#### 2.3.2. Bifurcation Envelope

For positioning systems comprising several antennas, the bifurcation curve changes dynamically depending on the paired times of arrival (TOAs) considered in each TDoA query. As discussed earlier, the system fails to estimate the position of an object within the concave regions of the bifurcation curves (i.e., those containing the anchors). In order to ensure a unique solution for any possible pairing, a so-called *bifurcation envelope* is defined which bounds all bifurcation curves on each anchor (e.g., one curve surrounding each anchor for three anchors and four curves for four anchors). In Figure 6c,d, the *flyable area* shaded in yellow is defined as the intersection of two areas:

1. The unique-solution area, defined as the intersection of all concave areas outside each green bifurcation envelope (i.e., not including anchors).  
2. The region with acceptable precision returned by the CRLB analysis (the convex hull).

The $i^{\mathrm{th}}$ transmitting anchor is represented by $m_{i}$, with $m_{1}$ being disregarded in Figure 6a. The centroids of triplets ($m_{i}$, $m_{j}$, $m_{k}$) are represented by $C_{ijk}$, while C is the collective centroid. Figure 6b,c shows the *flyable area* (shaded in yellow) and the convex hull of the four anchors (dotted magenta trapezoid, acceptable precision).

## 3. Filter Design

Thus far, we have analysed the *precision* and *failure* of UWB-based IPSs based on a pseudo-range multilateration algorithm with round-robin scheduling and signal TDoA. The aim in this section is to develop a filtering process to improve the *accuracy* of the system.

### 3.1. Proposed Filter Design

In our experimental setting, the object to be positioned is a Crazyflie 2.0 nano-quadcopter and the IPS is bitcraze’s Loco Positioning System [56]. This is setup already equipped with an Extended Kalman Filter (EKF) [57,58], which transforms raw sensor measurements into better estimates of the state of the drone (i.e., higher *precision*). The EKF developers note that the position estimates are affected by a measurement bias which appears to be non-uniform in space. In other words, the quadcopter is estimated to be in a position that is shifted from the actual one. To address this issue, we proposed that a debiasing filter be incorporated here after the built-in EKF.

In addition to a plain EKF, other practitioners may wish to incorporate additional filters to improve *precision*. An attempt to do this is discussed in Appendix C, although no meaningful *precision* improvement was observed with that particular set of filters.

### 3.2. Debiasing Filter

In this section, a filter is proposed and developed aiming to reduce systematic biases. Specifically, the aim of this *Debiasing Filter* (DF) is to increase the *accuracy* of the localisation of the drone by subtracting the expected bias from the measurements. Assuming that discrete distributions of variances and biases (see Section 4.1) have been obtained by statistical post-processing of consecutive position measurements, two major problems arise:

1. The bias values are available only at a limited set of points, and therefore they need to be interpolated to cover the continuous domain.
2. The bias to be subtracted from a measured position to obtain the actual one is a function of the actual position itself.

To address the first problem, a continuous model must be fitted to the limited data. This takes the form of a surface for 2D positioning (not necessarily defined on a regular grid) and a hypersurface in 4D space for 3D positioning. To address the second problem, a *bias map* must be built as a function of the measured rather than the actual positions.

**Figure 7:**
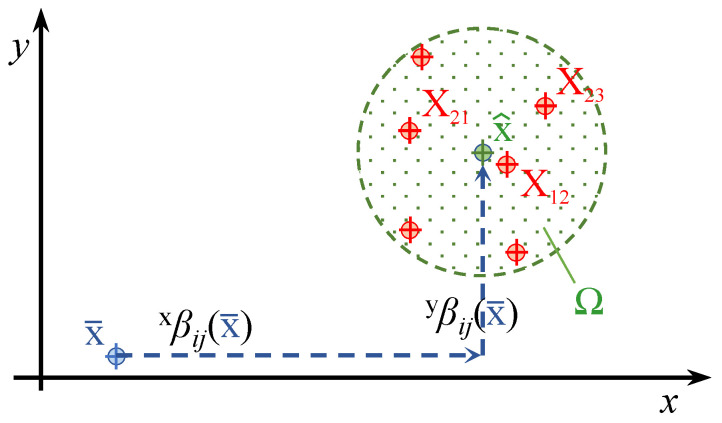
Expected position from the debiasing filter ($\hat{x}$) when applied to a measured posistion ($\bar{x}$) in 2D. The cloud of actual positions ($X_{ij}$) is constrained by the boundary Ω.

In order to explain the proposed debiasing process, we begin by defining the debiased measurement as $\hat{x}$, the measured posistion as $\bar{x}$, and the cloud of actual positions as $X_{ij}$, as shown in Equation (15) and Figure 7.

(15) $$\begin{array}{ll} \begin{array}{c} x=\left(x,y\right): \end{array} & Position\\ \bar{x}: & Measured\;x\\ X_{ij}=\left(X_{ij},Y_{ij}\right): & Cloud\;of\;actual\;x\;corresponding\;to\;(i,j)\;point\\ \hat{x}: & Debiased\;x \end{array}$$

Any measured position can be expressed as the sum of the actual position, a bias value (*b*), and a noise value (R), as shown in Equation (16) for independent *x* and *y* components. Note that both *b* and R depend on the actual position, where *p* stands for the variance.

(16) $$\begin{array}{c} \bar{x}=X_{ij}+{}^{x}b\left(X_{ij}\right)+R\left({}^{x}p\left(X_{ij}\right),x\right)\\ \bar{y}=Y_{ij}+{}^{y}b\left(X_{ij}\right)+R\left({}^{y}p\left(X_{ij}\right),x\right) \end{array}$$

Assuming R to be negligible, the real position can be obtained from the measurement $\bar{x}$ by simply subtracting the bias associated with the real position itself (see Equation (16) and Figure 7). It would be more practical to have the bias as a function of the measured positions rather than the actual positions. To this end, a set of bias measurements is obtained at a regular grid of points of known locations and added to the positions where they were measured, thereby obtaining an irregular grid. By fitting a model to the bias measurements associated with their corresponding points in the new grid, a *bias map* is obtained as a function of the measured rather than the actual postions: ${}^{x}\beta \left(\bar{x}\right)$ and ${}^{y}\beta \left(\bar{x}\right)$.

**Figure 8:**
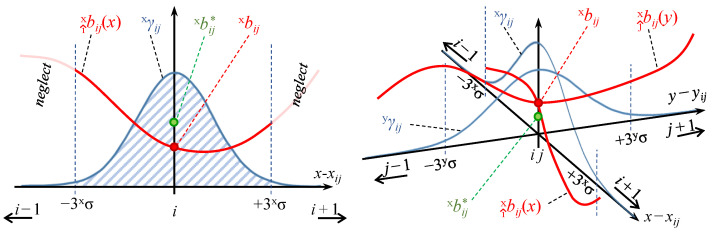
Representation of debiasing in 1D (left) and 2D (right).

For the purpose of the following derivation, continuous interpolating functions of the *x* and *y* biases, both along the $\hat{i}$ and $\hat{j}$ axes, have to be obtained (e.g., cubic splines). Therefore, from the biases of *x* measurements around any $x_{ij}$ position, two interpolating functions can be obtained, namely, ${}_{\hat{i}}^{x}b_{ij}$ and ${}_{\hat{j}}^{x}b_{ij}$ along the $\hat{i}$ and $\hat{j}$ axes, respectively. Likewise, ${}_{\hat{i}}^{y}b_{ij}$ and ${}_{\hat{j}}^{y}b_{ij}$ interpolating functions can be defined for the biases of *y* measurements. The aim is to write weighted averages of the biases around the estimation position in order to estimate the expected biases. However, instead of performing a surface integral, the average of two integrals in perpendicular directions is considered. Figure 8 (left) depicts the problem in the $\hat{i}$ direction for the bias of the measurement of the *x*-component of the position. Thus, the interpolated bias function ${}_{\hat{i}}^{x}b_{ij}$ multiplied by the weighting probability distribution ${}^{x}\gamma_{ij}$ is integrated in the *x* direction and normalised by the length of the considered interval. A coverage factor of k=3 is set, which means that approximately 99% (level of confidence) of the measurements of the real position ${{}^{r}x}_{ij}$ fall in the interval between ${\left(x-3{}^{x}\sigma \right)}_{ij}$ and ${\left(x+3{}^{x}\sigma \right)}_{ij}$, where ${}^{x}\sigma_{ij}$ is the standard deviation of the Gaussian distribution ${}^{x}\gamma_{ij}$. The same integral can be evaluated in the $\hat{j}$ direction and the two integral values can be averaged in order to obtain the corrected bias value of the *x*-component measurements, as shown in Equation (17) and Figure 8 (right). The same process can be applied for evaluating the corrected bias of the *y*-component measurements, as in Equation (18).

(17) $${}^{x}b_{ij}^{*}=\frac{1}{2}\int_{-3\cdot {}^{x}\sigma_{ij}}^{+3\cdot {}^{x}\sigma_{ij}}{}^{x}\gamma_{ij}\cdot {}_{\hat{i}}^{x}b_{ij}\cdot dx+\frac{1}{2}\int_{-3\cdot {}^{y}\sigma_{ij}}^{+3\cdot {}^{y}\sigma_{ij}}{}^{y}\gamma_{ij}\cdot {}_{\hat{j}}^{x}b_{ij}\cdot dy$$

(18) $${}^{y}b_{ij}^{*}=\frac{1}{2}\int_{-3\cdot {}^{x}\sigma_{ij}}^{+3\cdot {}^{x}\sigma_{ij}}{}^{x}\gamma_{ij}\cdot {}_{\hat{i}}^{y}b_{ij}\cdot dx+\frac{1}{2}\int_{-3\cdot {}^{y}\sigma_{ij}}^{+3\cdot {}^{y}\sigma_{ij}}{}^{y}\gamma_{ij}\cdot {}_{\hat{j}}^{y}b_{ij}\cdot dy$$

**Figure 9:**
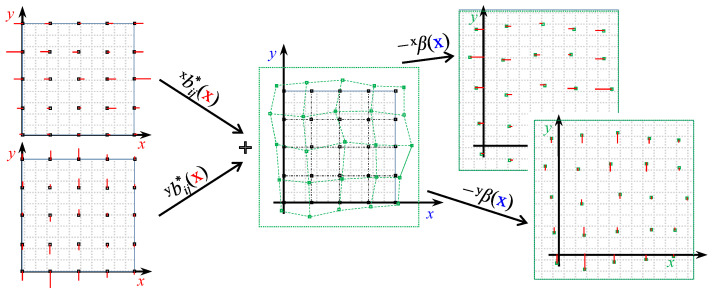
Diagram of derivation of debiasing functions in *x* direction ${}^{x}\beta \left(x\right)$ and *y* direction ${}^{y}\beta \left(x\right)$.

For the remaining derivations, refer to Figure 9. By rewriting the decomposition shown in Figure 7 and neglecting the noise component, the measured position is shifted from the original one approximately by the corrected weighted biases expressed in Equations (17) and (18) as shown in Equation (19).

(19) $$\begin{array}{c} {\bar{X}}_{ij}=X_{ij}+{}^{x}b_{ij}^{*}\\ {\bar{Y}}_{ij}=Y_{ij}+{}^{y}b_{ij}^{*} \end{array}$$

Therefore, while the original experimentally-obtained biases were distributed on a regular quadrangular grid $\left[{{}^{r}x}_{i,j}\;{{}^{r}y}_{i,j}\right]$, the new corrected biases $b^{*}$ can be distributed over a deformed grid $\left[{\bar{x}}_{i,j}\;{\bar{y}}_{i,j}\right]$. As shown in Equation (20), the two new corrected bias distributions of the *x* (${}^{x}m_{i,j}$) and *y* (${}^{y}m_{i,j}$) measurements can be interpolated, obtaining bias surfaces that are functions of the measured positions.

(20) $$\begin{array}{cc} {}^{x}m_{ij}={\left[\begin{array}{ccc} {\bar{X}}_{ij} & {\bar{Y}}_{ij} & {}^{x}b_{ij}^{*} \end{array}\right]}^{T} & \overset{interp.}{\to }{}^{x}\beta \left(x\right)\\ {}^{y}m_{ij}={\left[\begin{array}{ccc} {\bar{X}}_{ij} & {\bar{Y}}_{ij} & {}^{y}b_{ij}^{*} \end{array}\right]}^{T} & \overset{interp.}{\to }{}^{y}\beta \left(x\right) \end{array}$$

Finally, it is possible to subtract these new interpolating bias functions from the measured position in order to obtain a debiased measurement, as in Equation (21).

(21) $$\begin{array}{c} \hat{x}=x-{}^{x}\beta \left(x\right)\\ \hat{y}=y-{}^{y}\beta \left(x\right) \end{array}$$

Two examples of calibrated debiasing function fields can be found in Section 4.2 while the formulation of the Radial Basis Function Network (RBFN) used for interpolation of the debiasing values is explained in Appendix A.

## 4. Design of Experiments

### 4.1. IPS Bias Map Generation

**Figure 10:**
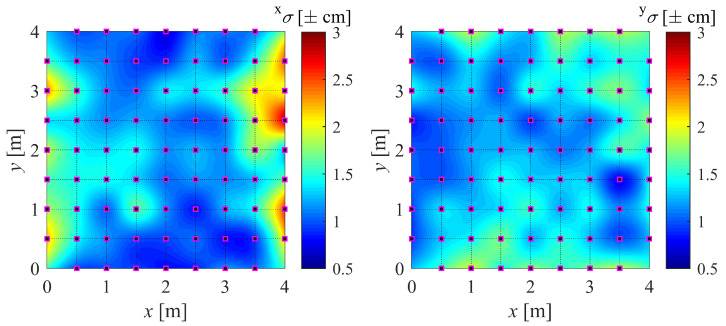
Precision map of *x* component ($\pm^{x}\sigma$) and *y* component ($\pm^{y}\sigma$) of position.

**Figure 11:**
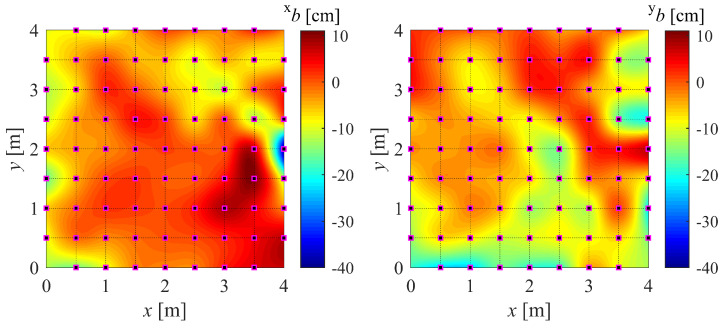
Accuracy map of *x* component ($\pm^{x}b$) and *y* component ($\pm^{y}b$) of position.

For simplicity, our version of the IPS (with built-in EKF + DF) is referred to as IPS-2, while the original version (only with built-in EKF) is referred to as IPS-1. In order to build the maps, a large number of measurements (N=700) are taken at a sampling frequency of 100 Hz while keeping the drone stationary for at least 30 s on each marker ($X_{ij}$). The drone is kept aligned with the *x* axis and parallel to the floor, as the effect of its attitude is not being investigated. Finally, the bias (*b*), standard deviation (σ), and mean squared error (MSE) are computed. For instance, their values in the *x* direction (superscript ${}^{x}$) are as in Equation (22), where $x^{\left(k\right)}$ is the $k^{\mathrm{th}}$ position measurement. The resulting maps can be found in Figure 10 and Figure 11.

(22) $$\begin{array}{c} {}^{x}b_{ij}=N^{-1}\sum_{k=1}^{N}x^{\left(k\right)}-X_{ij}\\ {}^{x}\sigma_{ij}={\left({{}^{x}\mathrm{MSE}}_{ij}-{{}^{x}b}_{ij}^{2}\right)}^{0.5}\\ {}^{x}{\mathrm{MSE}}_{ij}=N^{-1}\sum_{k=1}^{N}{\left(x^{\left(k\right)}-X_{ij}\right)}^{2} \end{array}$$

### 4.2. DF Calibration and Validation Setup

**Figure 12:**
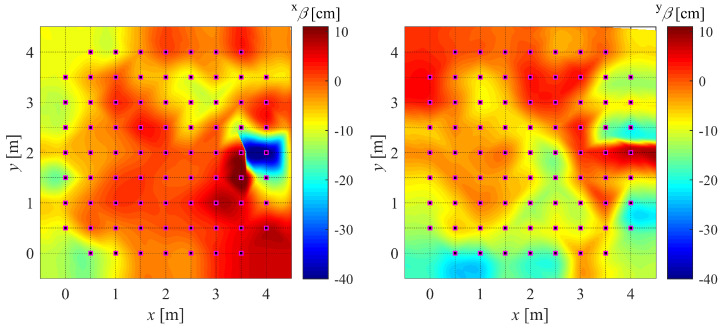
Debiasing function for measured *x* component (${}^{x}\beta$) and *y* component (${}^{y}\beta$) of position.

**Figure A2:**
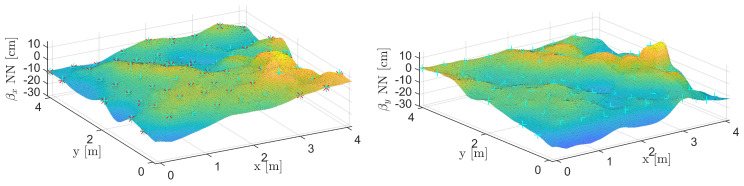
RBFN surface interpolating bias values of estimated position of the *x* (**left**) and *y* (**right**) components.

Using the obtained bias measurements, the debiasing filter for IPS-2 is calibrated using Radial Basis Function Networks (RBFN); refer to Appendix A for the formulation. This produces a final output similar to Figure 12 or Figure A2.

For validation, the estimates provided by IPS-2 are compared to those returned by IPS-1 at a predefined set of points (shown in Figure 1) not been previously used for calibration purposes. The variance and bias are evaluated to provide statistical insight into the performance of the newly developed filter.

### 4.3. DF Validation under Dynamic Setup Conditions

In addition to static validation, the estimates provided by IPS-1 and IPS-2 are further compared in a dynamic system with the vehicle cruising at different speeds.

The drone is mounted on a mobile stand which is constrained to move along an encoder rail (see Figure 2). In the frame of reference along the rail, the position (*s*) of the drone, and thus that of the optical sensor, is estimated as in Equation (23):

(23) $$s^{\left(k\right)}=\Delta s\cdot n^{\left(k\right)}$$

where Δs is the constant distance between consecutive pins (optical obstacles), *n* is the counted number of pins, and *k* is the measurement frequency at which the data from the sensors are recorded. Note that *n* is supposed to increase more slowly than *k*.

**Figure 13:**
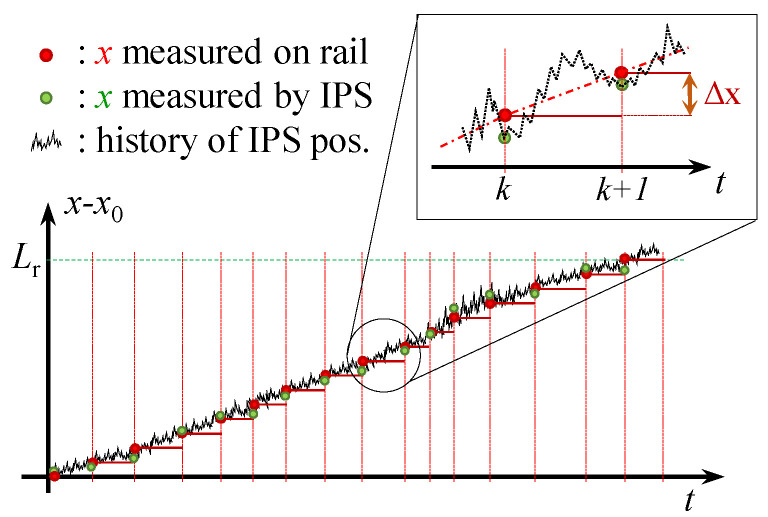
Visualisation of rail and IPS position measurements, where $L_{r}$ is the total length of the rail.

Figure 13 represents rail and IPS position measurements taken on a horizontal rail. The real position of the drone along the rail ($x_{r}^{\left(k\right)}$) in the inertial frame of reference can be obtained by projecting $s^{\left(k\right)}$ along the angle between the rail and the *x*-axis, $\theta_{r}$:

(24) $$\begin{array}{c} x_{r}^{\left(k\right)}=\left(x_{r}^{\left(k\right)},y_{r}^{\left(k\right)}\right)\\ x_{r}^{\left(k\right)}=s^{\left(k\right)}\cdot \cos\left(\theta_{r}\right)\;\mathrm{and}\;y_{r}^{\left(k\right)}=s^{\left(k\right)}\cdot \sin\left(\theta_{r}\right)\\ \theta_{r}=\mathrm{atan}\left(\frac{y_{b}-y_{a}}{x_{b}-x_{a}}\right) \end{array}$$

Every time a new pin is detected at time step *p*, the estimated position of both IPS-1 and IPS-2 ($x_{1}^{\left(k\right)}$,$x_{2}^{\left(k\right)}$) and the actual position on the rail are recorded as variables ξ and υ:

(25) $$\begin{array}{c} \mathrm{if}\;n^{\left(k\right)}>n^{\left(k-1\right)}\;\mathrm{then}:\\ \xi_{r}^{\left(p\right)}=x_{r}^{\left(k\right)}\;\upsilon_{r}^{\left(p\right)}=y_{r}^{\left(k\right)}\;\tau^{\left(p\right)}=t^{\left(k\right)}\\ \xi_{1}^{\left(p\right)}=x_{1}^{\left(k\right)}\;\upsilon_{1}^{\left(p\right)}=y_{1}^{\left(k\right)}\;\xi_{2}^{\left(p\right)}=x_{2}^{\left(k\right)}\;\upsilon_{2}^{\left(p\right)}=y_{2}^{\left(k\right)} \end{array}$$

In addition, the velocity estimations with IPS-1 and IPS-2 are compared to the discrete average velocity on the rail:

(26) $$\begin{array}{c} \nu_{x}^{\left(p\right)}=\frac{\Delta \xi_{r}^{\left(p\right)}}{\Delta \tau^{\left(p\right)}}\;\mathrm{and}\;\nu_{y}^{\left(p\right)}=\frac{\Delta \upsilon_{r}^{\left(p\right)}}{\Delta \tau^{\left(p\right)}}\\ \Delta \xi_{r}^{\left(p\right)}=\Delta s\cdot \cos\left(\theta_{r}\right)=\mathrm{const}.\\ \Delta \upsilon_{r}^{\left(p\right)}=\Delta s\cdot \sin\left(\theta_{r}\right)=\mathrm{const}.\\ \Delta \tau^{\left(p\right)}=\tau^{\left(p\right)}-\tau^{\left(p-1\right)} \end{array}$$

where $\Delta \tau^{\left(p\right)}$ is the time passed between the detection of the $p^{\mathrm{th}}$ and ${\left(p-1\right)}^{\mathrm{th}}$ pins.

Because the aim is to evaluate how well the IPS-2 performs with respect to IPS-1 at different crusing speeds of the drone, multiple measurements are required at different speeds. The recorded positional data are classified in different groups.

### 4.4. Square Path Experiment Setup

**Figure 14:**
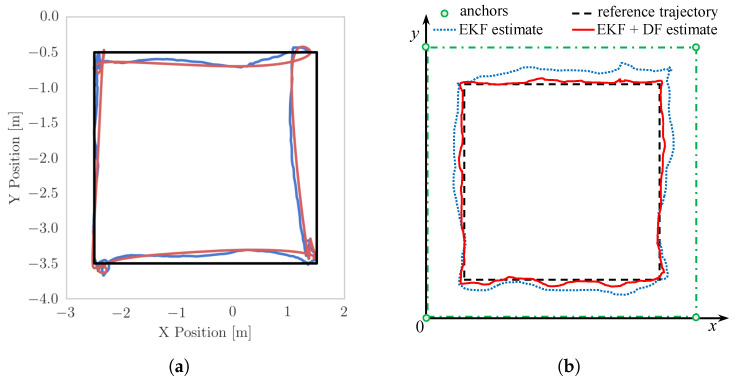
Square path experiments (**a**) from [57] and (**b**) carried out in this paper. (**a**) Drone flying in auto-pilot along a desired square path (black) (from [57]). Both the EKF estimate (blue) and the actual position (red) are shifted from the desired path. (**b**) Proposed experiment following a fixed square path (black). The EKF + DF estimation (red) is expected to be more accurate than the EKF-only estimation (blue).

The aim in this experiment is to partially reproduce the experiment with a drone following a square path [57] (Figure 14a). Because we want to investigate only the performance of the debiasing filter of IPS-2, we disable drone flight in favour of it being driven around by the mobile support along the square path. This decouples the performance of the debiasing filter from the control loop of the drone. The estimated positions of IPS-1 and IPS-2 are then compared. The expected result is depicted in Figure 14b.

## 5. Results and Discussion

### 5.1. Proof of Accuracy Improvement

**Figure 15:**
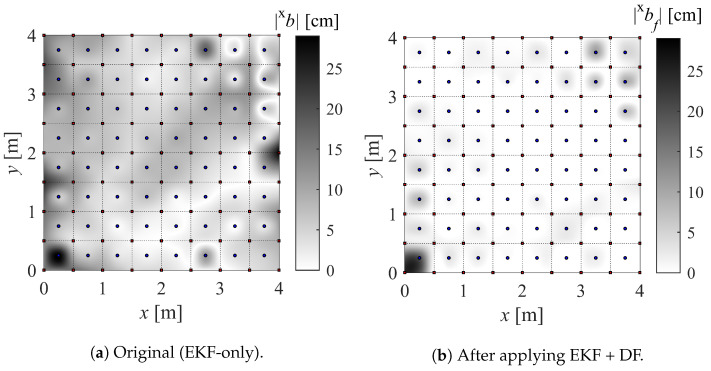
Absolute bias for *x*-direction measurements.

**Figure 16:**
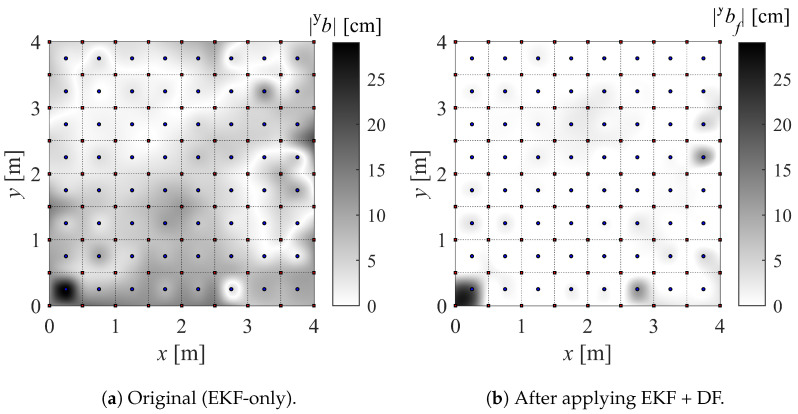
Absolute bias for *y*-direction measurements.

The results of the experiments in Section 4.2 are presented in this section along with the accompanying Figure 15 and Figure 16. In order to highlight the overall accuracy gain using the DF, the absolute value of the bias is represented. Note that the calibration points on the main grid are spaced 50 cm apart, while the validation mesh is staggered by 25 cm from the main calibration points.

The whiter areas in Figure 15 and Figure 16 correspond to more accurate areas. It can be seen that the contribution of the proposed DF is evident. It is noticeable that the DF fails to improve the accuracy in a few validation points, which means that the sampling points used for the mapping did not capture the gradient of the bias. A finer sampling mesh would most likely solve this issue. However, a compromise must be made between mapping refinement and the complexity of RBFN interpolation.

Another interesting aspect of the theoretical analysis previously carried out is manifested around the anchor positioned at (0,0). The points within the 50 cm radius around this anchor are undefined because these locations fall within the bifurcation envelope (refer to Section 2.3). No position can be measured in this area, and the DF is expected to fail.

### 5.2. Dynamic Validation of Debiasing

In this section, the results of the dynamic experiments in Section 4.3 and Section 4.4 are discussed. A reduction in the performance of the DF is expected, partly due to the intrinsic effects introduced by the positioning algorithm that are not addressed by the DF. Though less impressively than for the static case, the proposed DF improves the accuracy of the position estimates, as can be observed in Table 1 and Table 2. More precisely, Table 1 refers to the dynamic validation of the DF explained in Section 4.3, while Table 2 shows the statistics of the results of the square path experiment (Section 4.4).

**Figure 17:**
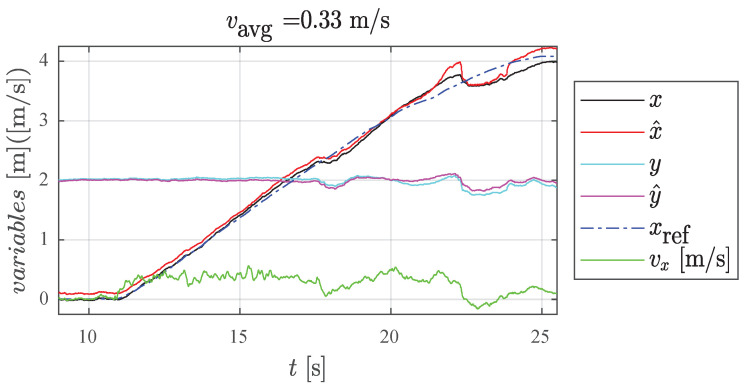
Dynamic experiment at average cruise velocity of 0.33 m/s with *x* spanning 0 m to 4 m at constant *y* = 2 m, where (x,y): position estimate with EKF-only; ($\hat{x},\hat{y}$): debiased position; ($x_{\mathrm{ref}}$): actual position on rail; and ($v_{x}$): estimated instantaneous velocity in *x*-direction.

An example of results from a single dynamic experiment are shown in Figure 17. This experiment was repeated ten times for each rail position in order to obtain the general trend of the IPS measurements.

**Figure 18:**
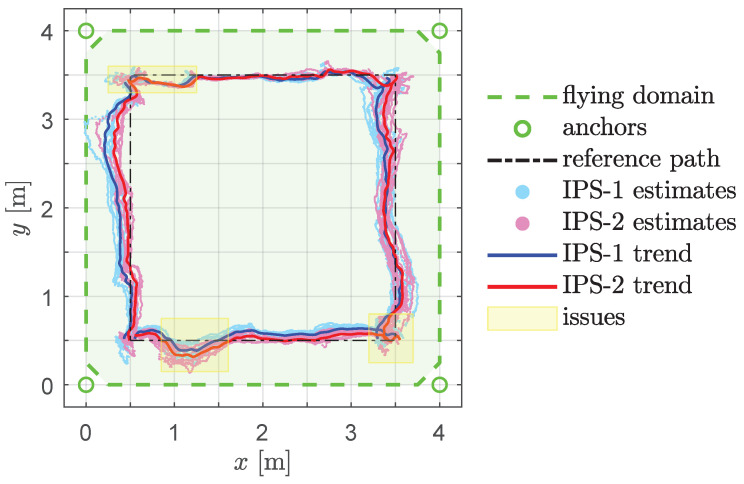
Square-path experiment results, with flying domain delimited by four anchors. The vehicle starts moving from (0.5,0.5) following the positive *x*-axis direction. IPS-1 uses EKF only, while IPS-2 uses EKF + DF. Problematic regions of the path are highlighted in yellow. The overall experiment shows a clear improvement when incorporating the proposed DF.

In Figure 17, note the dynamic *misbehaviour* of the IPS at around 22 s, which cannot be addressed by the proposed DF, although it could perhaps be handled by other filters (refer to Appendix C). Figure 18 presents a graphical representation of the overall results of the square-path experiment, while Table 2 presents a quantitative summary.

The Root Mean Square Errors (RMSEs) in Table 2 are computed by comparing the trendlines for each edge to the actual position of the drone on the rail at every time step. For instance, the RMSE_IPS-1_ on the bottom edge is obtained using the data cloud (cyan colour in Figure 18) of ten experiments on the edge from (0.5,0.5) to (3.5,0.5). The same dynamic issues that were pointed out in the validation experiment in Figure 17 persist in the square-path experiment (yellow regions in Figure 18). Assuming that the DF is insufficient to address the intrinsic problems of the IPS under study, we believe it may be useful to isolate this *misbehaviour* and provide statistics on the data unaffected by this (hence, the *raw* and *sel.* columns in Table 2).

## 6. Conclusions and Future Work

While the precision of IPSs is generally well studied, reasonably estimated, and provided by the manufacturer, failure and accuracy tend to be assessed poorly, if not plainly disregarded. In this paper, we carried out a comprehensive study of the *precision*, *failure*, and *accuracy* of 2D IPSs based on UWB technology and pseudo-range multilateration algorithm with round-robin scheduling using signal TDoA, in addition to developing a *debiasing filter* to *improve accuracy*. Although a number of aspects of this investigation are either general or can be generalised, the focus was on a specific setting of the IPS.

A theoretical study of the *precision* of the position estimates was performed based on a CRLB analysis taking into account the anisotropic radiation pattern of the anchors antennas. The *precision* is found to be higher within the convex hull of the anchors. Furthermore, visual inspection of the radiation pattern indicates that the orientation of the anchor antennas should probably be towards the centre of the domain.

A geometrical study of the two-dimensional positioning domain was carried out by bounding the areas in which the IPS is predicted to fail via bifurcation envelopes. The intersection between the convex hull and the region outside the bifurcation envelopes results in what we call the *flyable area*, within which the performance of the IPS is reliable.

The *accuracy* of the system was measured experimentally on a regular grid of points of known locations after applying a built-in EKF, thereby building a so-called *bias map* by fitting a Radial Basis Function Network (RBFN). In order to *improve accuracy*, a *debiasing filter* was developed by correcting the bias map to ensure that it depends on the measured rather than the actual positions. In this way, it can simply be subtracted from the measurements to debias them.

Our findings and developments were experimentally validated, with the IPS observed to *fail* near the anchors, *precision* found to be about ±3cm, and *accuracy* improved by about 15cm for static and by 5cm for dynamic measurements on average. The proposed method to define the *flyable area* and build the *precision maps*, *accuracy maps*, and *debiasing filter* is generalisable and repeatable for any other IPS that uses UWB technology and a multilateration algorithm based on the TDoA signal property. The numerical values reported here correspond to the specific IPS used in the experiments.

Future work might involve the generalisation of this study to 3D IPSs, the automation of the process to make it more easily applied in different settings, and the optimisation of the number, positioning, and orientation of the anchors in 3D to account for the slight anisotropy of the radiation pattern of the UWB module.

## Figures and Tables

**Table 1:** Representative results of dynamic validation. The RMSEs of an IPS with and another without DF (IPS-2 and IPS-1, respectively) are compared to demonstrate the accuracy improvement of the former. The average performance difference is shown in columns Δx and Δy. The average cruise velocity is shown in the last column.

			RMSE_*x*,avg_ [cm]			RMSE_*y*,avg_ [cm]			
**dir.**	x **[m]**	***y*** **[m]**	**IPS-1**	**IPS-2**	Δx	**IPS-1**	**IPS-2**	Δy	$v_{\mathrm{avg}}$
hor.	[0, 4]	1	12.7	6.8	5.9	10.0	7.9	2.1	0.58
hor.	[0, 4]	2	12.0	8.1	3.9	6.7	4.3	2.4	0.44
hor.	[0, 4]	3	12.6	8.0	4.6	9.3	8.0	1.3	0.43
ver.	1	[4, 0]	15.6	10.3	5.4	9.4	6.8	2.7	0.58
ver.	2	[4, 0]	10.3	8.0	2.3	15.8	10.1	5.7	0.51
ver.	3	[4, 0]	11.4	9.4	2.0	15.3	12.2	3.1	0.42

**Table 2:** Square-path experiment results. The RMSEs of an IPS with and another without DF (IPS-2 and IPS-1, respectively) are compared to show the accuracy improvement. The ‘raw’ heading refers to full data stream and the ‘sel.’ heading refers to the undamaged data stream (i.e., no misbehaviour). Average improvement with DF is shown by Δ.

				RMSE_IPS-1_ [cm]		RMSE_IPS-2_ [cm]		
**Edge**	**dir.**	***x*** **[m]**	***y*** **[m]**	**Raw**	**sel.**	**Raw**	**sel.**	Δ [cm]
bot	hor.	[0.5, 3.5]	0.5	9.2	9.5	7.5	4.7	4.8
right	ver.	3.5	[0.5, 3.5]	12.6	12.5	9.0	8.3	4.2
top	hor.	[3.5, 0.5]	3.5	6.0	5.5	5.7	4.6	0.9
left	ver.	0.5	[3.5, 0.5]	15.8	15.2	8.8	6.7	8.5

**Figure A1:**
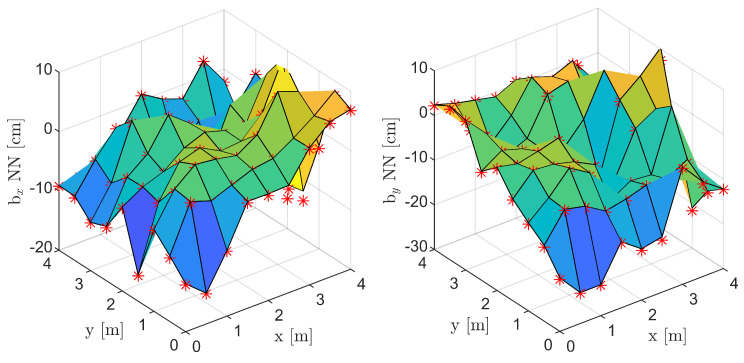
RBFN of bias values on marker points (red stars) for estimating the position of the *x* (**left**) and *y* (**right**) components.

**Figure A3:**
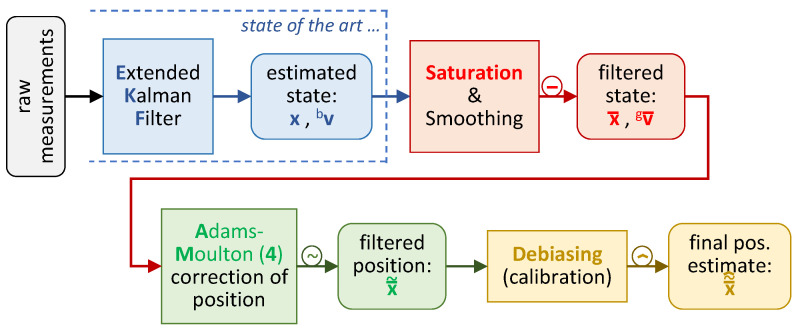
Proposed filtering process, consisting of four steps and returning estimates identified with their respective filter symbols: $\bar{x}$ stands for saturated, $\tilde{x}$ for dynamically corrected, and $\hat{x}$ for debiased. Superscripts ${}^{b}$ and ${}^{g}$ refer to the body frame and inertial frame, respectively.

**Figure A4:**
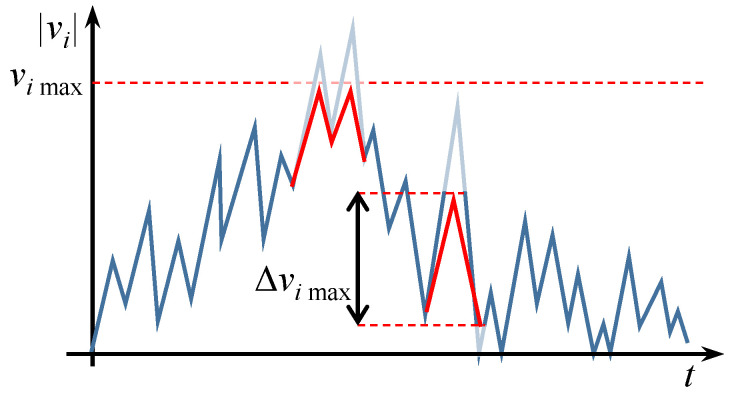
Representation of the presented saturation filter on velocity estimates in *i*th direction; *blue* shows the original EKF estimate and *red* shows the correction. Note that the measurements are discrete and represented by the peaks; the linear interpolation between measurements is only for visualisation purposes.

**Figure A5:**
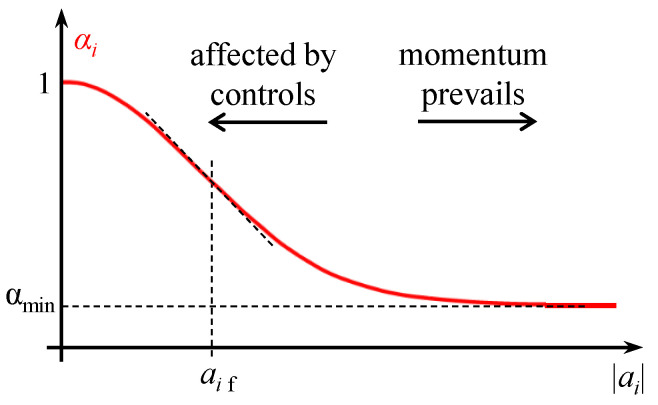
Weight function for averaging EKF state estimations and AM4 predictions; $v_{i\;f}$ is the flipping velocity, $v_{i\;\max}$ is the maximum expected speed, and $\alpha_{\min}$ is a calibration parameter.

## Abbreviations

The following abbreviations are used in this manuscript:

IPS	Indoor Positioning System
ToA	Time of Arrival
TDoA	Time Difference of Arrival
UWB	Ultra-Wideband
CRLB	Cramér–Rao Lower Bound
GNSS	Global Navigation Satellite System
GDoP	Geometric Dilution of Precision
CRLB	Cramér–Rao Lower Bound
EKF	Extended Kalman Filter
DF	Debiasing filter

## Appendix A. Radial Basis Function Network Implementation

In this section, the formulation of a Radial Basis Function Network (RBFN) is introduced for the interpolation of the debiasing distributions in both 2D environments (see Equation (20)). In the following equations, the change of variable expressed in Equation (A1) must be considered to ensure that the RBFN works on strictly positive values.

(A1) $$\begin{array}{c} b_{ij}={}^{x}b_{ij}^{*},\;{}^{y}b_{ij}^{*},\;\mathrm{or}\;{}^{z}b_{ij}^{*}\;\mathrm{and}\\ \mathrm{if}\;b_{\min}=\min\left\{b_{ij}\right\}<0\;\mathrm{then}\;b_{ij}=b_{ij}^{*}-b_{\min} \end{array}$$

The Gaussian activation function ($f_{ij}$) is defined on every marker ($X_{ij}$) that was previously used for mapping purposes. Hence, every marker represents a node in the network.

(A2) $$\begin{array}{c} f_{ij}\left(x\right)=c_{1}\cdot b_{ij}\cdot e^{-c_{2}\cdot d^{2}}\\ d^{2}={∥x-X_{ij}∥}^{2} \end{array}$$

While the constant $c_{2}$ can be uniquely defined for each node (see Equations (A3)–(A6)), the constant $c_{1}$ is used for calibration of the RBFN interpolation. The value of $c_{1}$ can be chosen between 0 and 1. By trial and error, the best acceptable value was found to be $c_{1}=0.5$.

(A3) $$\begin{array}{c} f_{ij}\left(X_{ij}\right)=c_{1}\cdot b_{ij}\\ f_{ij}\left(X_{mn}\right)=c_{1}\cdot b_{ij}\cdot e^{-c_{2}\cdot d_{mn}^{2}}=\frac{1-c_{1}}{8}b_{mn}\\ d_{mn}^{2}={∥X_{mn}-X_{ij}∥}^{2} \end{array}$$

(A4) $$\begin{array}{c} \ln\left(c_{3}\frac{b_{mn}}{b_{ij}}\right)=-c_{2}\cdot d_{mn}^{2}\\ c_{3}=\frac{1-c_{1}}{8\cdot c_{1}} \end{array}$$

(A5) $$c_{2,mn}=-\frac{1}{d_{mn}^{2}}\ln\left(c_{3}\frac{b_{mn}}{b_{ij}}\right)$$

The constant $c_{2,ij}\left(\theta \right)$ is a periodic function interpolating the values of $c_{2,mn}$ on a neighbourhood of eight surrounding points (in 2D).

(A6) $$\begin{array}{c} c_{2,ij}\left(\theta \right)=\mathrm{interp}\left(c_{2,mn},\theta_{mn}\right)\;\mathrm{periodic}\;\mathrm{in}\;\left[-\pi ,+\pi \right]\\ mn=[(i+1,j)\;(i+1,j+1)\;(i,j+1)\;(i-1,j+1)\;(i-1,j)\;\\ \left(i-1,j-1)\;(i,j-1)\;(i+1,j-1)\;(i+1,j)\right]\\ \mathrm{with}\;\theta_{mn}=\mathrm{atan}\left(\frac{Ymn-Yij}{X_{mn}-X_{ij}}\right) \end{array}$$

Therefore, after all the $c_{2,ij}\left(\theta \right)$ constants have been defined, the resulting RBFN (shown in Figure A1 and Figure A2) is as provided in Equation (A7).

(A7) $$\begin{array}{c} b^{\left(\mathrm{NN}\right)}\left(x\right)=b_{\min}+\sum_{i,j}^{N,M}f_{ij}\left(x\right)\\ f_{ij}\left(x\right)=c_{1}\cdot b_{ij}\cdot e^{-c_{2,ij}\cdot d_{ij}^{2}}\\ d_{ij}^{2}={∥x-X_{ij}∥}^{2}\\ c_{1}=0.5\\ c_{2,ij}=c_{2,ij}\left(\theta \right)\;\mathrm{periodic}\;\mathrm{in}\;\left[-\pi ,+\pi \right]\\ \mathrm{view}\;\mathrm{angle}\;\theta_{ij}=\mathrm{atan}\left(\frac{y-Yij}{x-X_{ij}}\right) \end{array}$$

Because the Gaussian activation functions are defined here such that they have influence only on the adjacent nodes, the debiasing filter (DF) code uploaded in the Crazyflie firmware takes account of this in order to optimise the performance in terms of real-time computation. Therefore, not all $f_{ij}\left(x\right)$ are computed at every time step, only those which are within a distance ($d_{ij}$) of 1 m from the measured position.

(A8) $$b^{\left(\mathrm{NN}*\right)}\left(x\right)=b_{\min}+\overset{N,M}{\sum_{i,j|d_{ij}<1m}}f_{ij}\left(x\right)$$

## Appendix B. Reference CRLB Analysis

Before proceeding with the analysis, the reference theory is used to set the background nomenclature. Considering an indoor positioning system consisting of only three anchors and using Time Difference of Arrival (TDoA) multilateration, the *pseudo-ranges* can be defined as the range differences between the node and each anchor:

(A9) $$\begin{array}{cc} \mathrm{pseudorange}: & \tau_{ij}\left(x\right)=d_{j}\left(x\right)-d_{i}\left(x\right)\\ \mathrm{range}: & d_{i}\left(x\right)=∥x-x_{i}∥\;\;\mathrm{and}\;\;i,j=1,2,3 \end{array}$$

where $d_{i}$ is the distance between the drone (x) and the $i^{\mathrm{th}}$ anchor position ($x_{i}$). According to [39], without loss of generality, the speed of propagation in the medium is considered to be 1 because the range is linearly dependent on the Time of Arrival (ToA). The value of $\tau_{ij}$ can be obtained by defining a TDoA mapping that transforms the two-dimensional space of the source location to a space of pseudo-ranges called the τ-plane, as suggested in [46]:

(A10) $$\begin{array}{cccc} \tau_{2}: & R^{2} & \to & R^{2}\\ \; & x & \to & \left(\tau_{12}\left(x\right),\tau_{13}\left(x\right)\right) \end{array}$$

Studying the TDoA map is crucial for the mathematical characterisation of the localisation problem. Considering an IPS that consists of a network of *N* anchors and one node (e.g., a drone), *M* measurements (number depending on the localisation algorithm) are obtained at every time step according to the frequency at which messages are sent from the anchors to the node. Every measurement is modeled as a normal distribution which is a function of both the real measurements and additive Gaussian noise. The standard deviation of the noise changes in space with the distance from any transmitting anchor. The collection of *M* measured pseudo-ranges $\hat{\tau }$ at time step *k* can be expressed as

(A11) $$\begin{array}{c} {\hat{\tau }}^{\left(k\right)}\in R^{M},\\ {\hat{\tau }}_{ij}∼N\left(\tau_{ij}\left(x\right),{\bar{\sigma }}_{ij}^{2}\right),\;{\bar{\sigma }}_{ij}=f\left(\sigma_{i},\sigma_{j}\right),\\ \tau_{ij}=∥x-x_{i}∥-∥x-x_{j}∥,\\ i,j\in \left\{1,\cdots ,N\right\}\;\mathrm{with}\;i\neq j, \end{array}$$

where ${\hat{\tau }}_{ij}$ is the individual pseudo-range measurement using the two ToAs of the node to the *i*th and *j*th anchors, and $\tau_{ij}$ is the real range difference. While the superscript ${}^{\left(k\right)}$ is omitted, it is implicitly inferred in further analyses. Please note that $\hat{\tau }$ is a column vector, not a matrix. Here, ${\hat{\tau }}_{ij}$ can have as many components as the binomial coefficient N3=N!3!(N−3)!. The functions evaluating the combined standard deviations are presented for the specific IPS under study in Section 2.

The well-known Cramér–Rao Lower Bound (CRLB) analysis is very useful for evaluating the precision of an unbiased IPS. Such analysis is based on the concept of the Fisher Information Matrix (FIM), which contains the likelihood of obtaining a correct measurement. The elements of the total FIM for the general positioning problem according to [48,52] are:

(A12) $$\begin{array}{c} {\mathrm{FIM}}_{ij}={\left(\frac{\partial \tau (x)}{\partial x_{i}}\right)}^{T}F_{\tau }^{-1}\left(x\right)\left(\frac{\partial \tau (x)}{\partial x_{j}}\right)\\ +\frac{1}{2}tr\left(F_{\tau }^{-1}\left(x\right)\frac{\partial F_{\tau }\left(x\right)}{\partial x_{i}}F_{\tau }^{-1}\left(x\right)\frac{\partial F_{\tau }\left(x\right)}{\partial x_{j}}\right) \end{array}$$

where *x* is the index of $\hat{\tau }$ measurements and tr(M) is the trace of a matrix M. Because each standard deviation is considered to be changing in space, i.e., $\sigma_{i}\left(x,y\right)$, the correction term (the second row in Equation (A12)) is acknowledged in the following analysis. The likelihood function, L, which describes the relative odds of obtaining the observed data h for all permissible values of the parameter x for a single measurement *h*, is:

(A13) $$L\left(\hat{h}|x\right)=\frac{1}{\sqrt{2\pi }\sigma \left(x\right)}e^{\left(-\frac{1}{2\sigma^{2}\left(x\right)}{\left(\hat{h}-h\left(x\right)\right)}^{2}\right)}$$

In our positioning problem, h(x) is the range, or similarly the ToA, and the parameter x is the node position. The standard deviation of this distribution is again σ. Considering the Fisher Information Matrix (FIM) in terms of likelihood [48,52], the logarithmic likelihood has to be considered:

(A14) $$\ell \left(\hat{h}|x\right)=\ln\left(\frac{1}{\sqrt{2\pi }\sigma \left(x\right)}\right)-\frac{1}{2}\frac{{\left(\hat{h}-h\left(x\right)\right)}^{2}}{\sigma^{2}\left(x\right)}$$

Hence, the total FIM for the location, considering that each standard deviation changes in space ($\sigma_{i}=\xi \left(x,y\right)$), is as follows:

(A15) $${\mathrm{FIM}}_{ij}={\left(\frac{\partial h(x)}{\partial x_{i}}\right)}^{T}F_{\tau }^{-1}\left(x\right)\left(\frac{\partial h(x)}{\partial x_{j}}\right)+\frac{1}{2}tr\left(F_{\tau }^{-1}\left(x\right)\frac{\partial F_{\tau }\left(x\right)}{\partial x_{i}}F_{\tau }^{-1}\left(x\right)\frac{\partial F_{\tau }\left(x\right)}{\partial x_{j}}\right)$$

where tr(M) is the trace of the matrix *M*.

Here, $F_{\tau }$ is the information matrix of the selected set of TDoA measurements. Suppose that the TDoA protocol requires M measurements; then, $F_{\tau }$ is

(A16) $$F_{\tau }={\left[F_{ij}\right]}_{M\times M}$$

Using an efficient unbiased estimator, it is proven [52] that $F_{\tau }$ is the measurement covariance matrix:

(A17) $$F_{\tau }={\left[E\left[\left({\hat{\tau }}_{ij}-{\bar{\tau }}_{ij}\right)\left({\hat{\tau }}_{kp}-{\bar{\tau }}_{kp}\right)\right]\right]}_{M\times M}$$

where the indices i,j,k,p depend on the selected scheduling.

As an example, if all the TDoA measurements are performed while keeping anchor 1 as the reference (as in all the studies found in the literature), all $\tau_{1i}$ values would be correlated with the standard deviation in measurements of anchor 1 ($\sigma_{1}^{2}$). Therefore, the resulting FIM for the measurement set $\tau_{1}=\left\{\tau_{12},\tau_{13},\tau_{14}\right\}$ is as in Equation (A18).

(A18) $$F_{\tau }=\left[\begin{array}{ccc} s_{1}+s_{2} & s_{1} & s_{1}\\ s_{1} & s_{1}+s_{3} & s_{1}\\ s_{1} & s_{1} & s_{1}+s_{4} \end{array}\right]$$

where $s_{i}=\sigma_{i}^{2}$.

## Appendix C. Initial Filter Design

As discussed in Section 3, the IPS in our experimental setting is already equipped with an Extended Kalman Filter (EKF). The EKF developers note that the position estimates are affected by a non-uniform bias.

Our original idea was to use multiple filtering layers to enhance both precision and accuracy (see Figure A3). While the EKF is the first filter by default, the remaining ones may be applied in any order. The reason the *debiasing filter* (refer to Section 3.2) is applied last is that the bias values can change dramatically throughout the flying area, while the precision values do not (and have smaller magnitudes). Hence, we deemed it better to work on a more precise estimate to avoid selecting the wrong value of the bias. The proposed filtering process is defined as follows:

1. Extended Kalman Filter
2. Saturation (and artificial smoothing)
3. Correction of position via fourth-order Adams–Moulton (AM4) method
4. Debiasing filter

Unfortunately, precision was not noticeably enhanced. Therefore, only the EKF and the *Debiasing Filter* discussed in Section 3 were implemented for the experiments carried out in this paper (see Section 4 and Section 5). Nonetheless, the mathematical formulations of the other filtering layers are included below to support future work.

### Appendix C.1. Saturation and Smoothing Filter

The saturation filter limits the eventual estimation overshoots of both velocity and position. Two types of velocity overshoots are considered, namely, a maximal overestimation and a maximum allowed time derivative. For example, each component of the estimated velocity vector is limited by a maximum velocity value that can be different in the three directions. For instance, the horizontal velocity components (in *x* and *y* direction) can be limited by the maximum cruise speed, while the vertical velocity (in *z* direction) can be at maximum two times the free-fall speed. Moreover, the time derivative of the velocity is limited by the expected maximum acceleration. For simplicity of analysis, the derivatives in all directions have been limited by the same amount, though this may not be the case in practice. The effect of the presented saturation filter on a casual sequence of velocity estimations is depicted in Figure A4. The filter acts analogously on a sequence of position estimates, i.e., Equations (A23)–(A25).

We start by defining the scalar variation of velocity (${}^{g}\Delta v^{\left(k\right)}$) as the modulus of the difference between the EKF-estimated velocity at the current time ${}^{\left(k\right)}$ and the filtered velocity at the previous time step ${}^{\left(k-1\right)}$:

(A19) $${}^{g}\Delta v^{\left(k\right)}=∥\Delta^{g}v^{\left(k\right)}∥=∥{}^{g}v^{\left(k\right)}-{}^{g}{\bar{v}}^{\left(k-1\right)}∥$$

This value should not exceed the maximum allowed speed variation (limited by the maximum acceleration, $a_{\max}$). Therefore, the inequality (A20) can be used to formulate the ceiling of the velocity variation ($\Delta^{g}{\bar{v}}^{\left(k\right)}$) in Equation (A21):

(A20) $${}^{g}\Delta v^{\left(k\right)}⩽\Delta t\cdot a_{\max}$$

(A21) $$\Delta^{g}{\bar{v}}^{\left(k\right)}=\min\left(\Delta t\cdot a_{\max}\cdot {{}^{g}\Delta v^{\left(k\right)}}^{-1}\;,\;1\right)\cdot \Delta^{g}v^{\left(k\right)}$$

At this point, every velocity component has to be limited by the maximum allowed speeds ($v_{i\;\max}$). The components of the final filtered velocity vectors (${}^{g}{\bar{v}}^{\left(k\right)}$) are therefore defined as follows:

(A22) $$\begin{array}{c} {}^{g}{\bar{v}}_{i}^{\left(k\right)}=\min\left({}^{g}{\bar{v}}_{i}^{\left(k-1\right)}+\Delta^{g}{\bar{v}}_{i}^{\left(k\right)}\;,\;v_{i\;\max}\right)\\ v_{\max}=\left[\begin{array}{ccc} v_{1\;\max} & v_{2\;\max} & v_{3\;\max} \end{array}\right] \end{array}$$

The same filtering procedure can be applied to the position estimates, although in this case only the time derivative is limited. As before, the inequality (A20) can be used to define the variation of each $i^{\mathrm{th}}$ component (i=1,2,3) of the filtered position $\Delta {\bar{x}}^{\left(k\right)}$ expressed in Equation (A24).

(A23) $$\begin{array}{c} \Delta x_{i}^{\left(k\right)}⩽\Delta t\cdot v_{i\;\max} \end{array}$$

where $\Delta x^{\left(k\right)}=∥\Delta x^{\left(k\right)}∥=∥x^{\left(k\right)}-{\bar{x}}^{\left(k-1\right)}∥$

(A24) $$\Delta {\bar{x}}_{i}^{\left(k\right)}=\min\left(\Delta t\cdot v_{i\;\max}\cdot {\Delta x^{\left(k\right)}}^{-1},1\right)\cdot \Delta x_{i}^{\left(k\right)}$$

Finally the filtered estimation of the position can be defined as follows:

(A25) $${\bar{x}}^{\left(k\right)}={\bar{x}}^{\left(k-1\right)}+\Delta {\bar{x}}^{\left(k\right)}$$

Embodied in this filtering step is the smoothing filter that aims to artificially reduce the oscillations of the measurements by averaging the history of n+1 measurements. The average is weighted such that the contribution of the last measurement (${\bar{x}}^{\left(k\right)}$) is more important:

(A26) $$\begin{array}{c} {\bar{x}}_{s}^{\left(k\right)}={\left(1-q\right)}^{n}\cdot {\bar{x}}^{\left(k-n\right)}+\sum_{n-1}^{l=0}q\cdot {\left(1-q\right)}^{l}\cdot {\bar{x}}^{\left(k-l\right)}\\ q=q_{\%}/100 \end{array}$$

where $q_{\%}$ is the percentage influence of the last measurement.

### Appendix C.2. Adams–Moulton Filter

The main idea of Adams–Moulton dynamic prediction is to correct the EKF estimations, which consider only the measurements at the current time, using a prediction that takes into account the previous history of estimations. Hence, it is a multistep measurement filter. Usual multi-step predictor–corrector schemes consist of the combination of an explicit predictor, e.g., Adams–Bashforth (AB) of order *n*, with an implicit corrector, e.g., Adams–Moulton (AM) of order *m*, in order to take advantage of the prediction of the derivative function provided by AB(n). For the exception of a presented correction filter, AM can be used directly. In (A27), an AM scheme of fourth order is defined for predicting the position ${\bar{x}}_{\mathrm{AM}4}^{\left(k\right)}$ via the use of filtered velocity estimates at the current and previous four time steps. A higher order AM scheme could be selected to consider a longer history of estimates.

(A27) $$\begin{array}{c} {\bar{x}}_{\mathrm{AM}4}^{\left(k\right)}={\bar{x}}^{\left(k-1\right)}+\frac{\Delta t}{720}\cdot \left(251\cdot {}^{g}{\bar{v}}^{\left(k\right)}+646\cdot {}^{g}{\bar{v}}^{\left(k-1\right)}\right.\\ \left.-264\cdot {}^{g}{\bar{v}}^{\left(k-2\right)}+106\cdot {}^{g}{\bar{v}}^{\left(k-3\right)}-19\cdot {}^{g}{\bar{v}}^{\left(k-4\right)}\right) \end{array}$$

One aspect that is considered by the EKF estimates and neglected by AM4 prediction is the effect that the control command input has on the state. For instance, the EKF takes into account the given thrust input. Intuitively, at low cruise speeds, the drone’s dynamics are very affected by control input; thus, the EKF estimate is more important, while at high cruise speed the drone dynamics at short period are mostly a direct effect of the stored momentum of the flying body, and move by inertia. Therefore, a vectorial weighting funcion α is defined here to ensure that the filtered correction of the estimated position follows this concept. The formulation of the newly filtered position ${\tilde{\bar{x}}}^{\left(k\right)}$ is shown in (A28), while the shape of the *i*th weight component function (A29) is shown in Figure A5.

(A28) $${\tilde{\bar{x}}}^{\left(k\right)}=\alpha ⊙{\bar{x}}^{\left(k\right)}+\left(1-\alpha \right)⊙{\bar{x}}_{\mathrm{AM}4}^{\left(k\right)}$$

where ⊙ is the component-wise multiplication operation; thus, given two vectors a and b, the resulting vector components are $c_{i}=a_{i}\cdot b_{i}$, with i=1,2,3.

(A29) $$\alpha_{i}=\exp\left(-\frac{1}{2}{\left(\frac{{\bar{a}}_{i}^{\left(k\right)}}{a_{i\;f}}\right)}^{2}\right)\left(1-\alpha_{\min}\right)+\alpha_{\min}$$
